# Prevalence of Self-Reported Sleep Problems Among People With Diabetes in the United States, 2005-2008

**Published:** 2012-03-22

**Authors:** Laura Plantinga, Madhu N. Rao, Dean Schillinger

**Affiliations:** Department of Epidemiology, Rollins School of Public Health, Emory University. At the time of the study, Ms Plantinga was also affiliated with the Center for Vulnerable Populations, San Francisco General Hospital, San Francisco, California, and the Division of General Internal Medicine, University of California, San Francisco, California; University of California, San Francisco, California; San Francisco General Hospital, San Francisco, California; University of California, San Francisco, California; and California Department of Public Health, Sacramento, California

## Abstract

**Introduction:**

Sleep problems, including insomnia, apnea, and restless legs syndrome, are common, burdensome, and under-recognized in the United States. We sought to examine the association of sleep problems with diabetes among community-dwelling US adults.

**Methods:**

We examined self-reported sleep problems in 9,848 adults (aged ≥20 y) participating in the National Health and Nutrition Examination Survey 2005 through 2008. Sleep problem information was elicited via validated questionnaire. Diabetes was defined by self-reported diagnosis or glycohemoglobin of 6.5% or higher. Multivariable logistic regression with US population-based weighting was used to obtain adjusted odds ratios (ORs) and 95% confidence intervals (CIs) for various sleep problems by diabetes status.

**Results:**

Sleep problems were common (>90% for any problem; 10%-40% for individual problems) overall, and people with diabetes were more likely than those without diabetes to report multiple problems (mean, 3.1 vs 2.5, respectively, *P* < .001). After adjustment for potential confounders (including demographics, body mass index, cardiovascular and kidney disease, and alcohol use), restless legs symptoms (OR, 1.40; 95% CI, 1.12-1.78), sleep apnea (OR, 1.45; 95% CI, 1.06-1.98), and nocturia (OR, 1.51; 95% CI, 1.22-1.87) were all positively associated with diabetes status.

**Conclusion:**

Diabetes is associated with a higher risk of sleep problems, including not only sleep apnea but also inadequate sleep, excessive sleepiness, leg symptoms, and nocturia, independent of body mass index. Clinicians should be aware of the high prevalence of sleep problems among their patients with diabetes and should consider screening and treatment, which may improve patients' quality of life.

## MEDSCAPE CME

Medscape, LLC is pleased to provide online continuing medical education (CME) for this journal article, allowing clinicians the opportunity to earn CME credit.

This activity has been planned and implemented in accordance with the Essential Areas and policies of the Accreditation Council for Continuing Medical Education through the joint sponsorship of Medscape, LLC and Preventing Chronic Disease. Medscape, LLC is accredited by the ACCME to provide continuing medical education for physicians.

Medscape, LLC designates this Journal-based CME activity for a maximum of 1 **AMA PRA Category 1 Credit(s)™**. Physicians should claim only the credit commensurate with the extent of their participation in the activity.

All other clinicians completing this activity will be issued a certificate of participation. To participate in this journal CME activity: (1) review the learning objectives and author disclosures; (2) study the education content; (3) take the post-test with a 70% minimum passing score and complete the evaluation at www.medscape.org/journal/pcd (4) view/print certificate.

**Release date: March 22, 2012; Expiration date: March 22, 2013**

### Learning Objectives

Upon completion of this activity, participants will be able to:

- Describe the overall prevalence of sleep problems among persons with diabetes as based on 2005-2008 NHANES data.
- Describe factors modifying the prevalence of sleep problems among persons with diabetes as based on 2005-2008 NHANES data.
- Describe the prevalence of specific sleep problems among persons with diabetes as based on 2005-2008 NHANES data.

**CME EDITOR**

Nancy Saltmarsh, Editor, *Preventing Chronic Disease*. Disclosure: Nancy Saltmarsh has disclosed no relevant financial relationships.

**CME AUTHOR**

Laurie Barclay, MD. Freelance writer and reviewer, Medscape, LLC. Disclosure: Laurie Barclay, MD, has disclosed no relevant financial relationships. 

**AUTHORS**

Disclosures: Laura Plantinga, ScM; Madhu N. Rao, MD; and Dean Schillinger, MD, have disclosed no relevant financial relationships.

## Introduction

One-third of US adults report inadequate sleep (1). Sleep problems are associated with cardiovascular disease (2), mental health problems (3), motor vehicle accidents (4), and overall poor quality of life (5). The direct and indirect costs of sleep problems are substantial (6). Despite the individual and societal burden of sleep problems, most (80%-90%) remain undiagnosed (7).

Both diabetes (8) and sleep problems (5) are highly correlated with poor quality of life. People with diabetes may also be at higher risk for sleep problems relative to the general population because of common risk factors for diabetes and sleep problems, including advanced age, obesity, and treatments for and complications of common comorbid diseases (eg, depression, cardiovascular disease). Additionally, diabetes-specific complications, such as neuropathy, could directly interfere with sleep. However, because of low rates of detected sleep disorders (7), most people with diabetes who have sleep problems are likely to go untreated. Thus, estimating the prevalence of such undiagnosed sleep problems can help determine whether these problems are a public health concern.

Although there are more than 80 recognized sleep disorders (9), studies of diabetes and sleep have focused mainly on sleep apnea (10). Additionally, most studies of diabetes and sleep problems have lacked comparisons to the general population and have depended on the diagnosis of sleep disorder. We sought to examine the association of self-reported sleep problems — which are likely to be more prevalent than diagnosed sleep disorders — with diabetes among community-dwelling US adults, using data from the National Health and Nutrition Examination Survey (NHANES) 2005 through 2008. We focused on the most common sleep problems: sleep apnea, insomnia, nocturia, and leg symptoms.

## Methods

### Study design

NHANES is conducted by the Centers for Disease Control and Prevention (CDC) and consists of a standardized in-home interview followed by a physical examination at a mobile examination center. NHANES uses representative samples of noninstitutionalized US civilian residents. We limited our analysis to NHANES 2005 through 2008 adult (≥20 y) participants who responded to the sleep questionnaire and completed the examination (n = 9,848). NHANES 2005 through 2006 and 2007 through 2008 reported response rates of 77.4% and 75.4% for the examined participants, respectively. All participants gave written informed consent. The National Center for Health Statistics Research Ethics Review Board approved the protocol.

### Measurements

**Questionnaire**

Interviewers administered a questionnaire pertaining to sleep habits and sleep-related problems (www.cdc.gov/nchs/data/nhanes/nhanes_05_06/sp_slq_d.pdf), including items from 2 validated instruments: the Sleep Heart Health Study Sleep Habits Questionnaire (11) and the Functional Outcomes of Sleep Questionnaire (12). We have described individual items previously (13). Nocturia was an item on a separate kidney conditions – urology questionnaire (www.cdc.gov/nchs/data/nhanes/nhanes_05_06/sp_kiq_d.pdf). Participants self-reported demographics (age, sex, race/ethnicity), socioeconomic status and health care access (education, income, and health insurance coverage), and health status and conditions (diagnosis of diabetes, hypertension, and cardiovascular disease [CVD]; smoking and alcohol use; and depressive symptoms from the Patient Health Questionnaire-9 [PHQ-9] [14]) via questionnaire. The interviewer recorded prescription medications from the bottles provided by the participant.

**Examination and laboratory measurements**

Laboratory personnel performed glycohemoglobin measurements using a high-performance liquid chromatography system and assessed fasting plasma glucose concentration by a hexokinase method. Laboratory personnel also measured serum and urine creatinine by the modified kinetic method of Jaffe and urine albumin using solid-phase fluorescence immunoassay. Examination personnel recorded anthropomorphic measurements (weight and height, used to calculate body mass index [BMI]) and blood pressure (≥3 auscultatory measurements).

### Definitions

**Sleep problems**

We used inadequate sleep, sleep deprivation, daytime sleepiness, and sleeping pill use as markers of insomnia. We defined inadequate sleep as less than 7 total hours of sleep, according to National Sleep Foundation guidelines (15) (Box); we defined excess sleep as 9 or more hours per night. We defined severe sleep deprivation by sleep onset latency 5 minutes or less (16). We considered daytime sleepiness and sleeping pill use to be frequent if they were reported "often" or more (≥5 times/mo). We defined apnea by frequent self-reported apnea, snorting, or gasping, or diagnosed sleep apnea. We defined nocturia as reported urination of 2 or more times per night. We included frequent self-reported leg jerks or cramps, or diagnosed restless legs syndrome as leg symptoms (Box).

**Box. Definition and Measurement of Sleep Problems, NHANES, 2005-2008**

**Table:** 

**Box. Definition and Measurement of Sleep Problems, NHANES, 2005-2008**			
**Problem**	**Questionnaire Item^a^**	**Possible Responses**	**Definition**
Inadequate sleep	How much sleep do you usually get at night on weekdays or workdays?	Any number of hours with upper limit of 12	<7 h
Severe sleep deprivation	How long does it usually take you to fall asleep?	Any number of minutes with upper limit of 60	≤5 min
Frequent daytime sleepiness	In the past month, how often did you feel excessively or overly sleepy during the day?	Never, rarely (1 time/mo), sometimes (2-4 times/mo), often (5-15 times/mo), almost always (16-30 times/mo)	Often/almost always
Frequent sleeping pill use	In the past month, how often did you take sleeping pills or other medication to help you sleep?		
Apnea	In the past 12 months, how often did you snort, gasp, or stop breathing while you were sleeping?	Never, rarely (1-2 nights/wk), occasionally (3-4 nights/wk), frequently (≥5 nights/wk)	Often/almost always or yes and sleep apnea
	Have you ever been told by a doctor or other health professional that you have a sleep disorder?	Yes, no	
	What was the sleep disorder?	Sleep apnea, insomnia, restless legs, other	
Nocturia^b^	During the past 30 days, how many times per night did you most typically get up to urinate, from the time you went to bed at night until the time you got up in the morning?	Any number with upper limit of 5	≥2 episodes/ night
Leg symptoms	In the past month, how often did you have leg jerks while trying to sleep?	Never, rarely (1 time/mo), sometimes (2-4 times/mo), often (5-15 times/mo), almost always (16-30 times/mo)	Often/almost always or yes and restless legs
	In the past month, how often did you have leg cramps while trying to sleep?		
	Have you ever been told by a doctor or other health professional that you have a sleep disorder?	Yes, no	
	What was the sleep disorder?	Sleep apnea, insomnia, restless legs, other	

Abbreviation: NHANES, National Health and Nutrition Examination Survey.

a Adapted by NHANES from the Sleep Heart Study Sleep Habits Questionnaire.

b From kidney conditions – urology questionnaire of NHANES.

**Diabetes**

We defined diabetes by self-report (answer of yes to the question, "Other than during pregnancy, have you ever been told by a doctor or other health care provider that you have diabetes or sugar diabetes?") or glycohemoglobin of 6.5% or more (17). We did not include fasting plasma glucose of 126 mg/dL or more in the main definition because of the small sample of fasting morning participants (n = 4,770).

**Other definitions**

We defined self-reported diseases by answers of yes to the question, "Have you ever been told by a doctor or other health professional that you have [disease or condition]." We defined self-reported CVD by an answer of yes to any of coronary artery disease, angina, myocardial infarction, stroke, or congestive heart failure. We defined hypertension by self-report or by measured blood pressure of 140/90 mm Hg or higher. We defined depressive symptoms by at least 5 positive responses on the PHQ-9, along with reported functional impairment. We calculated estimated glomerular filtration rate (eGFR) according to the Modification of Diet in Renal Disease equation for isotope dilution mass spectrometry traceable creatinine (18). We defined albuminuria by a single urinary albumin:creatinine ratio of 30 mg/g or more.

### Statistical analysis

We compared selected characteristics for participants with and without diabetes using χ^2^ tests for categorical variables and *t* tests for continuous variables. We calculated prevalence of sleep problems by diabetes classification. We used multivariable logistic regression to obtain adjusted odds ratios (ORs) and 95% confidence intervals (CIs); covariates were chosen both for evidence of confounding and for parsimony. We performed sensitivity analyses with different definitions of diabetes among those not reporting sleeping pill use, with further covariates, and stratified by possible effect modifiers. All analyses were performed using Stata version 10.0 (StataCorp LP, College Station, Texas) to account for study design weights, strata, and primary sampling units.

## Results

### Characteristics of US population by diabetes status

Participants with diabetes were older and more likely to be non-Hispanic black, to have no high school diploma, and to be insured than those without diabetes (Table 1). Relative to their counterparts, those with diabetes were less likely to be daily smokers, drank fewer alcoholic beverages per week, and were more likely to have obesity, depression, hypertension, cardiovascular disease, albuminuria, and reduced kidney function and to use diuretics (Table 1).

### Prevalence of sleep problems

Overall, 93% of the adult US population reported at least 1 of the problems examined (Table 2). Leg symptoms (41%), inadequate sleep (37%), severe sleep deprivation (29%), and nocturia (25%) were the most commonly reported problems. While frequent sleeping pill use and apnea were less commonly reported, nearly 1 in 10 reported either. Those with diabetes were more likely than those without diabetes to report multiple sleep problems (mean no. of problems, 3.1 vs 2.5; Figure).

**Figure.**
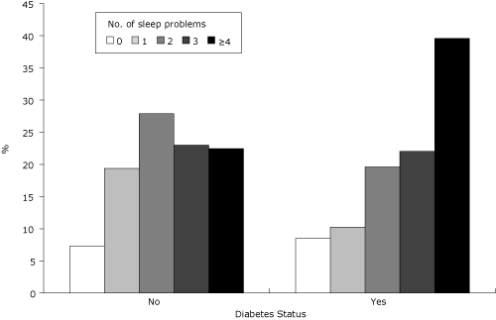
Number of reported sleep problems by diabetes status. The mean number of problems was 2.5 for the no-diabetes group and 3.1 for the diabetes group. Differences between the 2 groups were significant at *P* < .001 in terms of both percentage distribution and mean number of problems (calculated by using χ^2^ and *t* tests, respectively).

**Table:** 

Diabetes	% Reporting Sleep Problems, by No. of Sleep Problems				
	0	1	2	3	≥4
No	7.29	19.37	27.87	23.01	22.46
Yes	8.5	10.24	19.63	22.05	39.57

### Association of sleep problems with diabetes

Diabetes was associated with increased odds of inadequate sleep, frequent daytime sleepiness, restless legs symptoms, sleep apnea, and nocturia (Table 3). These associations were generally robust after adjustment for confounding factors. The magnitude of the associations of sleep apnea and, to a slightly lesser extent, inadequate sleep with diabetes was attenuated by adjustment for BMI but remained significant. The associations of diabetes with daytime sleepiness and inadequate sleep were rendered nonsignificant with the final adjustment for alcohol use.

Sensitivity analyses examining the association of sleep problems with diabetes defined only by glycohemoglobin showed similar results, except that the association between glycohemoglobin and sleep apnea was fully explained by BMI (data not shown). Results with diabetes defined by self-report, glychohemoglobin, and fasting plasma glucose were nearly identical to our primary analyses. When diabetes was examined by severity category (no diabetes, diabetes with glycohemoglobin <7.5%, diabetes with glycohemoglobin >7.5%), apnea, leg symptoms, daytime sleepiness, and nocturia (but not inadequate sleep) all showed greater odds with increasing severity in a significant, graded fashion. Diabetes duration was significantly associated with the same problems; risk increased 20% to 30% per 10 years since diagnosis.

Furthermore, the associations of diabetes with sleep problems were also similar among the subset of the population not reporting sleeping pill use (data not shown). Adjustment for other possible confounders generally did not substantially change the results: waist circumference was associated with sleep apnea (per 1-cm increase: OR, 1.03; 95% CI, 1.02-1.05), but its addition to the model did not alter the association of diabetes with apnea (OR, 1.41; 95% CI, 1.02-1.95). Similarly, the association of diabetes with nocturia was not affected by either pulse pressure (OR, 1.48; 95% CI, 1.20-1.84) or systolic blood pressure (OR, 1.48; 95% CI, 1.19-1.83). Higher income was generally associated with lower prevalence of sleep problems (with the exception of apnea, which showed the opposite association for the highest income category); however, its addition to the models did not affect the association of diabetes with sleep problems.

Stratified analyses showed that the association of sleep apnea, leg symptoms, and nocturia with diabetes was strongest among participants younger than 60 years old compared to those 60 or older (leg symptoms: OR, 1.94 vs 1.24; *P* = .006; apnea: OR, 3.52 vs 1.59; *P* < .001; and nocturia: OR, 3.09 vs 1.55; *P* < .001) (data not shown). The association of leg symptoms with diabetes was stronger among women than men (OR, 1.65 vs 1.28; *P* = .04). This effect modification by age and sex was not significant for any other sleep problem, and race/ethnicity did not modify the effect of these associations.

## Discussion

We found that sleep problems are highly prevalent in the United States. More than 90% of NHANES respondents reported any examined sleep problem and 10% to 40% reported any given problem. Inadequate sleep, frequent daytime sleepiness, apnea, leg symptoms, and nocturia were all more common among those with diabetes than those without diabetes. These associations persisted after adjustment for conditions that could contribute to poor sleep and were generally strongest among respondents who were younger and female. Although frequent sleeping pill use and severe sleep deprivation were both common, the prevalence of these problems did not differ by diabetes status.

The association of diabetes with sleep apnea, which was independent of obesity — a risk factor for both conditions — has been shown previously (10), as has our observed association between diabetes and restless legs symptoms (19), which may have neuropathic origins or be related to commonly used medications such as second-generation antidepressants and antihistamines. However, less is known about other sleep problems among people with diabetes. We found that nocturia and excessive sleepiness were also independently associated with diabetes. Nocturia was the most common sleep problem reported in this population, and the association with diabetes persisted even after adjustment for diuretic use. Nocturia is often not considered among the common sleep problems, but it may have deleterious effects on both sleep and quality of life (20), which was not assessed in this study. Inadequate sleep and excess sleepiness are similarly under-recognized (21) and detrimental to physical and mental health.

Although we have shown that diabetes is independently associated with increased risk of several sleep problems, the cross-sectional study design precludes causal inference, despite the magnitude and dose-response nature of the observed associations. Additionally, while we found that duration of diabetes was positively associated with increased risk of apnea, nocturia, leg symptoms, and daytime sleepiness — suggesting a possible temporal relationship between diabetes and subsequent sleep problems — we cannot definitively establish the directionality of the association. Previous research examining potential causal links between diabetes and sleep problems has been inconclusive. For example, a small study showed that forced sleep deprivation in healthy young men led to decreased leptin levels and increased appetite (22); another cross-sectional analysis of participants in the Sleep Heart Health Study showed that impaired glucose tolerance was more common among people with habitual sleep restriction (23), suggesting that sleep problems may lead to diabetes via physiologic mechanisms. However, the prevalence of sleep problems among patients with type 1 diabetes was found to be similar to that seen in type 2 diabetes (24). Given the difference in etiology, these results suggest that diabetes may also lead to sleep problems.

Other limitations of note include the self-report of sleep problems, which is subject to recall and detection bias. Data for neuropathy or chronic pain, pruritis, caffeine use, neck circumference, and 24-hour blood pressure were not available in the surveys analyzed. Restless legs symptoms may not be limited to leg cramps or jerks. Sleep studies were not performed. Although we were able to show that adjustment for income did not affect the association between diabetes and sleep problems, we did not have data on poverty-related stressors. Such stressors are thought to increase the "allostatic load" and both interfere with sleep and increase risk for chronic diseases such as diabetes (25).

However, our study also augments previous research on diabetes and sleep problems in several ways. It is a large, nationally representative study examining a comprehensive range of sleep problems, regardless of diagnosis. We were able to compare prevalence among people with diabetes to that of similar people without diabetes, to determine whether diabetes had an independent effect on sleep problems after adjustment for other factors strongly related to both diabetes and sleep, including age, sex, obesity, cardiovascular disease, depression, kidney disease, alcohol use, and income.

In conclusion, people with diabetes are more likely to have sleep problems than their counterparts of the same age, sex, and race/ethnicity without diabetes, regardless of several behaviors and comorbid conditions that could interfere with sleep. Because sleep problems and diabetes are both associated with poor quality of life (5,8), further research is warranted. Such efforts should examine the possibly dual directionality of the association of diabetes and sleep problems and explore possible causal inference and associated outcomes such as quality of life, ideally in a longitudinal cohort. Our results suggest that diabetes patients should be screened for these treatable sleep problems (sleep apnea, insomnia, restless legs, and nocturia). Recognition and treatment of sleep problems, particularly in the disproportionately affected younger and female diabetes populations, could considerably improve quality of life and, possibly, clinical outcomes among these patients.

## Figures and Tables

**Evaluation:** 

**1. The activity supported the learning objectives.**				
Strongly Disagree				Strongly Agree
1	2	3	4	5
**2. The material was organized clearly for learning to occur.**				
Strongly Disagree				Strongly Agree
1	2	3	4	5
**3. The content learned from this activity will impact my practice.**				
Strongly Disagree				Strongly Agree
1	2	3	4	5
**4. The activity was presented objectively and free of commercial bias.**				
Strongly Disagree				Strongly Agree
1	2	3	4	5

**Table 1.** Characteristics of the US Adult Population, Overall and by Diabetes Status, NHANES, 2005-2008

Characteristic	Total % (95% CI)^a^ (N = 9,848)	Diabetes Status, % (95% CI)		

		No Diabetes (n = 8,424)	Diabetes (n = 1,424)	*P* Value^b^
**Demographics**				
**Mean age, y (95% CI)**	46.7 (45.8-47.5)	45.5 (44.7-46.4)	58.9 (57.7-60.1)	<.001
**Sex**				
Male	48.0 (47.1-48.9)	47.9 (47.0-49.0)	48.5 (44.8-52.2)	.77
Female	52.0 (51.1-52.9)	52.1 (51.1-53.0)	51.5 (47.8-55.2)	
**Race/ethnicity^c^**				
Non-Hispanic white	71.4 (66.5-75.8)	72.4 (67.7-76.6)	62.6 (54.2-70.3)	<.001
Non-Hispanic black	10.7 (8.3-13.7)	9.9 (7.6-12.8)	17.7 (13.6-22.8)	
Mexican American	8.2 (6.5-10.2)	8.1 (6.5-10.1)	8.8 (6.5-11.8)	
**Socioeconomic status**				
**Education**				
<High school	18.9 (16.8-21.2)	17.7 (15.6-20.0)	29.1 (26.0-32.5)	<.001
≥High school	81.1 (78.8-83.2)	82.3 (80.0-84.4)	70.9 (67.6-74.0)	
**Annual household income, $**				
<20,000	15.4 (13.9-17.1)	14.6 (13.1-16.2)	22.8 (19.5-26.4)	<.001
20,000-44,999	27.8 (25.4-30.2)	27.1 (24.6-29.7)	33.8 (30.2-37.5)	
45,000-74,999	24.2 (22.5-26.0)	24.6 (22.9-26.4)	21.0 (17.7-24.9)	
≥75,000	32.6 (29.5-35.9)	33.8 (30.5-37.2)	22.3 (19.1-25.9)	
**Health insurance**				
Not insured	19.0 (16.9-21.2)	19.6 (17.6-21.9)	12.8 (10.4-15.7)	<.001
Insured	81.0 (78.8-83.1)	80.3 (78.1-82.4)	87.2 (84.3-89.6)	
**Clinical status**				
**Smoking**				
Every day	19.8 (17.8-21.9)	20.3 (18.1-22.6)	15.8 (13.3-18.7)	.01
Sometimes/not at all	80.2 (78.1-82.2)	79.8 (77.4-81.9)	84.2 (81.3-86.7)	
**Mean body mass index, kg/m^2^ (95% CI)**	28.5 (28.3-28.8)	28.1 (27.7-28.3)	32.9 (32.3-33.5)	<.001
**Depression**				
Symptomatic by PHQ-9	5.1 (4.5-5.9)	4.9 (4.2-5.7)	7.2 (5.4-9.4)	.02
Not symptomatic by PHQ-9	94.9 (94.1-95.6)	95.1 (94.3-95.8)	92.9 (90.7-94.6)	
**Hypertension^d^**				
Yes	41.8 (39.8-43.8)	38.4 (36.4-40.5)	71.8 (68.8-74.6)	<.001
No	58.2 (56.2-60.2)	61.6 (59.5-63.6)	28.2 (25.4-31.2)	
**Cardiovascular disease^e^**				
Yes	8.7 (7.8-9.6)	6.5 (5.8-7.4)	27.2 (24.0-30.7)	<.001
No	91.3 (90.4-92.2)	93.5 (92.7-94.2)	72.8 (69.3-76.0)	
**Albuminuria status**				
UACR ≥30 mg/g	8.0 (7.2-8.8)	6.1 (5.5-6.9)	23.6 (20.6-26.8)	<.001
UACR <30 mg/g	92.0 (91.2-92.8)	93.9 (93.2-94.5)	76.4 (73.2-79.4)	
**Reduced kidney function**				
eGFR <60 mL/min/1.73 m^2^	8.3 (7.2-9.5)	7.0 (5.9-8.3)	19.5 (16.9-22.4)	<.001
eGFR ≥60 mL/min/1.73 m^2^	91.7 (90.5-92.8)	93.0 (91.7-94.1)	80.5 (77.6-83.1)	
**Mean alcoholic intake, no. of drinks/wk (95% CI)**	3.8 (3.5-4.1)	4.0 (3.7-4.4)	2.1 (1.7-2.6)	<.001
**Diuretic use**				
Yes	6.0 (5.3-6.7)	5.4 (4.7-6.1)	11.3 (9.7-13.2)	<.001
No	94.0 (93.3-94.7)	94.6 (93.9-95.3)	88.7 (86.8-90.3)	

Abbreviations: NHANES, National Health and Nutrition Examination Survey; CI, confidence interval; PHQ-9, patient health questionnaire, 9-item; UACR, urinary albumin:creatinine ratio; eGFR, estimated glomerular filtration rate.

a Values are percentages except where indicated by the word "mean."

b Calculated by using χ^2^ tests for categorical variables and analysis of variance for continuous variables.

c Other race/ethnicity category (including other Hispanic, Asian, Pacific Islander, and Native American) not shown because of within-category heterogeneity, but respondents in "other" category are included in all analyses.

d Self-reported or measured blood pressure ≥140/90 mm Hg.

e Self-reported.

**Table 2.** Prevalence of Selected Sleep Problems Among US Adults, NHANES, 2005-2008

**Problem**	%
Any problem	92.6
Leg symptoms	41.1
Inadequate sleep	37.0
Severe sleep deprivation	28.7
Nocturia	24.5
Frequent daytime sleepiness	18.6
Frequent sleeping pill use	9.1
Apnea	8.9

Abbreviation: NHANES, National Health and Nutrition Examination Survey.

**Table 3.** Odds of Sleep Problems for People With Diabetes, NHANES, 2005-2008

**Problem^a^^,^ b**	OR (95% CI) vs No Diabetes
**Inadequate sleep**	
Unadjusted	1.33 (1.16-1.52)
Adjusted	1.16 (0.97-1.38)
**Severe sleep deprivation**	
Unadjusted	1.05 (0.86-1.28)
Adjusted	1.09 (0.84-1.42)
**Frequent daytime sleepiness**	
Unadjusted	1.38 (1.15-1.65)
Adjusted	1.26 (0.98-1.63)
**Frequent sleeping pill use**	
Unadjusted	1.60 (1.20-2.12)
Adjusted	1.26 (0.95-1.68)
**Leg symptoms**	
Unadjusted	1.68 (1.43-1.99)
Adjusted	1.40 (1.12-1.78)
**Sleep apnea**	
Unadjusted	2.49 (2.01-3.08)
Adjusted	1.45 (1.06-1.98)
**Nocturia**	
Unadjusted	2.91 (2.41-3.51)
Adjusted	1.51 (1.22-1.87)

Abbreviations: NHANES, National Health and Nutrition Examination Survey; OR, odds ratio; CI, confidence interval.

a See Box in Methods for definitions.

b Data were adjusted for age, sex, race/ethnicity, body mass index (continuous), cardiovascular disease, depression, albuminuria, kidney function, diuretic use, and alcohol use.

## Post-Test Information

To obtain credit, you should first read the journal article. After reading the article, you should be able to answer the following, related, multiple-choice questions. To complete the questions (with a minimum 70% passing score) and earn continuing medical education (CME) credit, please go to http://www.medscape.org/journal/pcd. Credit cannot be obtained for tests completed on paper, although you may use the worksheet below to keep a record of your answers. You must be a registered user on Medscape.org. If you are not registered on Medscape.org, please click on the "Register" link on the right hand side of the website to register. Only one answer is correct for each question. Once you successfully answer all post-test questions you will be able to view and/or print your certificate. For questions regarding the content of this activity, contact the accredited provider, CME@medscape.net. For technical assistance, contact CME@webmd.net. American Medical Association's Physician's Recognition Award (AMA PRA) credits are accepted in the US as evidence of participation in CME activities. For further information on this award, please refer to http://www.ama-assn.org/ama/pub/category/2922.html. The AMA has determined that physicians not licensed in the US who participate in this CME activity are eligible for *AMA PRA Category 1 Credits™*. Through agreements that the AMA has made with agencies in some countries, AMA PRA credit may be acceptable as evidence of participation in CME activities. If you are not licensed in the US, please complete the questions online, print the AMA PRA CME credit certificate and present it to your national medical association for review.

## Post-Test Questions

### Article Title: Prevalence of Self-Reported Sleep Problems Among People With Diabetes in the United States, 2005-2008

**CME Questions**

1. On the basis of the study of 2005-2008 NHANES data by Dr. Plantinga and colleagues, which of the following statements about the overall prevalence of sleep problems among persons with diabetes is **most likely **correct?
  1. About half of persons in the survey reported having any sleep problem.
  2. Among persons with diabetes, the mean number of sleep problems was about 2.
  3. The study proves that diabetes causes sleep problems.
  4. People with diabetes are more likely to have sleep problems than are persons of the same age, sex, and race/ethnicity without diabetes.
2. On the basis of the study of 2005-2008 NHANES data by Dr. Plantinga and colleagues, which of the following statements about factors modifying the prevalence of sleep problems among persons with diabetes is **most likely** correct?
  1. Adjustment for conditions that could contribute to poor sleep abolished the observed associations between sleep problems and diabetes.
  2. The observed associations between sleep problems and diabetes were strongest among older respondents.
  3. The observed associations between sleep problems and diabetes were stronger in men than in women.
  4. Duration of diabetes was positively associated with increased risk for specific sleep problems.
3. Your patient is a 56-year-old man with type 2 diabetes. On the basis of the study of 2005-2008 NHANES data by Dr. Plantinga and colleagues, which of the following statements about the prevalence of specific sleep problems would **most likely** be correct?
  1. Adjusted risk for restless legs symptoms is increased by about 40% compared with someone without diabetes.
  2. He is at increased risk for frequent sleeping pill use compared with someone without diabetes.
  3. He is at increased risk for severe sleep deprivation compared with someone without diabetes.
  4. After adjustment for diuretic use, risk for nocturia is not increased compared with persons without diabetes.

## References

[1] Colten HR, Altevogt BM (2006). Sleep disorders and sleep deprivation: an unmet public health problem.

[2] Ayas NT, White DP, Manson JE, Stampfer MJ, Speizer FE, Malhotra A, Hu FBl (2003). A prospective study of sleep duration and coronary heart disease in women. Arch Intern Med.

[3] Strine TW, Chapman DP (2005). Associations of frequent sleep insufficiency with health-related quality of life and health behaviors. Sleep Med.

[4] Connor J, Norton R, Ameratunga S, Robinson E, Civil I, Dunn R (2002). Driver sleepiness and risk of serious injury to car occupants: population based case control study. BMJ.

[5] Verster JC, Pandi-Perumal SR, Streiner D (2008). Sleep and quality of life in clinical medicine.

[6] Skaer TL, Sclar DA (2010). Economic implications of sleep disorders. PharmacoEconomics.

[7] Young T, Evans L, Finn L, Palta M (1997). Estimation of the clinically diagnosed proportion of sleep apnea syndrome in middle-aged men and women. Sleep.

[8] Laiteerapong N, Karter AJ, Liu JY, Moffet HH, Sudore R, Schillinger D (2011). Correlates of quality-of-life in older adults with diabetes: The Diabetes & Aging Study. Diabetes Care.

[9] American Academy of Sleep Medicine (2005). The international classification of sleep disorders: diagnostic and coding manual.

[10] Rasche K, Keller T, Tautz B, Hader C, Hergenc G, Antosiewicz J (2010). Obstructive sleep apnea and type 2 diabetes. Eur J Med Res.

[11] O'Connor GT, Lind BK, Lee ET, Nieto FJ, Redline S, Samet JM (2003). Variation in symptoms of sleep-disordered breathing with race and ethnicity: the Sleep Heart Health Study. Sleep.

[12] Weaver TE, Laizner AM, Evans LK, Maislin G, Chugh DK, Lyon K (1997). An instrument to measure functional status outcomes for disorders of excessive sleepiness. Sleep.

[13] Plantinga LC, Lee K, Stevens L, Saran R, Yee J, Gillespie B (2011). Association of sleep-related problems with chronic kidney disease in the United States, 2005-2008. Am J Kidney Dis.

[14] Kroenke K, Spitzer RL, Williams JB (2001). The PHQ-9: validity of a brief depression severity measure. J Gen Intern Med.

[15] National Sleep Foundation (2009). How much sleep do we really need?.

[16] Dement WC, Vaughn C (2009). The promise of sleep: a pioneer in sleep medicine explores the vital connection between health, happiness, and a good night's sleep.

[17] American Diabetes Association (2011). Standards of medical care in diabetes — 2011. Diabetes Care.

[18] Selvin E, Manzi J, Stevens LA, Van Lente F, Lacher DA, Levey AS (2007). Calibration of serum creatinine in the National Health and Nutrition Examination Surveys (NHANES) 1988-1994, 1999-2004. Am J Kidney Dis.

[19] Cuellar NG, Ratcliffe SJ (2008). Restless legs syndrome in type 2 diabetes: implications to diabetes educators. Diabetes Educ.

[20] Ancoli-Israel S, Bliwise DL, Norgaard JP (2011). The effect of nocturia on sleep. Sleep Med Rev.

[21] Roth T, Bogan RK, Culpepper L, Doghramji K, Doghramji P, Drake C (2010). Excessive sleepiness: under-recognized and essential marker for sleep/wake disorder management. Curr Med Res Opin.

[22] Spiegel K, Tasali E, Penev P, Van Cauter (2004). Brief communication: sleep curtailment in healthy young men is associated with decreased leptin levels, elevated ghrelin levels, and increased hunger and appetite. Ann Intern Med.

[23] Gottlieb DJ, Punjabi NM, Newman AB, Resnick HE, Redline S, Baldwin CM, Nieto FJ (2005). Association of sleep time with diabetes mellitus and impaired glucose tolerance. Arch Intern Med.

[24] Borel AL, Benhamou PY, Baguet JP, Halimi S, Levy P, Mallion JM, Pépin JL (2010). High prevalence of obstructive sleep apnoea syndrome in a type 1 diabetic adult population: a pilot study. Diabet Med.

[25] Van Cauter E, Spiegel K (1999). Sleep as a mediator of the relationship between socioeconomic status and health: a hypothesis. Ann NY Acad Sci.

